# Contarini’s Syndrome: Bilateral Pleural Effusions Due to Different Etiologies

**DOI:** 10.7759/cureus.52051

**Published:** 2024-01-10

**Authors:** Tatsunori Shizuku, Miyu Osawa, Izumi Kitagawa, Yasuharu Tokuda

**Affiliations:** 1 General Internal Medicine, Shonan Fujisawa Tokushukai Hospital, Kanagawa, JPN; 2 Internal Medicine, Muribushi Project for Teaching Hospitals, Urasoe, JPN

**Keywords:** respiratory care, contarini’s syndrome, bilateral pleural effusions, emergency medicine, geriatric medicine

## Abstract

Contarini's syndrome is a condition in which the occurrence of bilateral pleural effusions is attributed to different causes for each side. The decision to perform bilateral thoracentesis can be challenging for clinicians, particularly in elderly patients with multiple comorbidities. A 75-year-old Asian man with a past medical history of dementia and dysphagia presenting with dyspnea was brought to our emergency department. Imaging studies revealed bilateral pleural effusions and multiple costal fractures. The results of bilateral thoracentesis indicated an exudate pleural effusion in the right lung and a hemorrhagic pleural effusion in the left lung. Given the results, we determined the etiology of the right pleural effusion to be a parapneumonic effusion resulting from aspiration pneumonia, while the left hemorrhagic pleural effusion was due to costal fractures. After initiating treatment with antibiotics and placement of bilateral drainage tubes, the patient’s condition improved remarkably. This case underscores the importance of considering bilateral thoracentesis, particularly in geriatric patients.

## Introduction

Contarini’s syndrome, characterized by bilateral pleural effusions resulting from different etiologies, occurs in approximately 5% of patients with bilateral pleural effusions [1-3]. Some studies suggest risk factors, including elderly age and immunocompromised status, are associated with Contarini’s syndrome [1,2]. Although there are no specific criteria regarding the timing for considering bilateral thoracentesis in patients with bilateral pleural effusions, bilateral thoracentesis should be considered promptly for patients with Contarini’s syndrome to improve their clinical conditions.

## Case presentation

**Figure 1:**
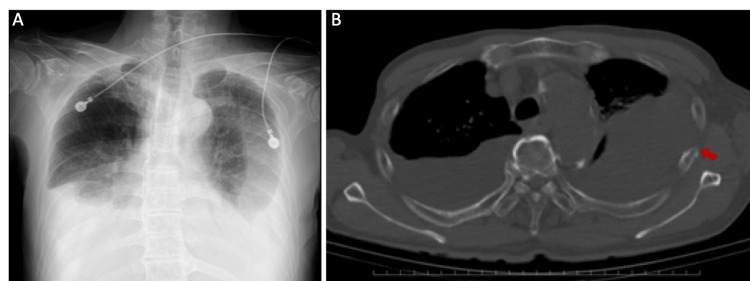
Imaging studies Chest x-ray shows bilateral pleural effusions (A). Computed tomography of the lung demonstrates a size discrepancy between the effusions and indicates a costal fracture (red arrow) (B).

A 75-year-old Asian man with a past medical history of dementia and dysphagia presenting with dyspnea was brought to our emergency department after caregivers in the nursing home found him lying down on the floor two hours earlier. Taking a medical history from him was impossible due to decreased cognitive function. In addition to dementia and dysphagia, his medical history included schizophrenia and epilepsy, treated with antipsychotics and antiepileptics. He was bedridden, but was able to sit on the wheelchair with help. In vital signs, his oxygen saturation was 58% with room air. Blood pressure was 150/90 mmHg, heart rate was 99 beats per minute with sinus rhythm on the ECG, and body temperature was 36.5 °C. Respiratory rate was 16 per minute. Oxygen saturation recovered to 95% with 10 L/min with an oxygen reservoir mask. The patient was disoriented. Physical examination was positive for pitting edema in lower extremities, coarse crackles on lungs, increased tactile fremitus on both lungs, bruises on the left chest and conjunctival pallor, but negative for subcutaneous crepitus, jugular venous distention, heart murmurs and paradoxical breathing. He appeared to grimace intensely when the bruises were palpated. The results of the arterial blood gas analysis with room air indicated a pH of 7.38, a partial pressure of oxygen of 43 mmHg, a partial pressure of carbon dioxide of 43 mmHg, and a bicarbonate level of 26 mEq/L upon admission. In the blood tests, the hemoglobin level was 8.9 g/dl, and the creatinine level was 0.72 mg/dl. The albumin level was 3.0 g/dl. The brain natriuretic peptide (BNP) level was normal. Inflammatory markers, such as leukocytosis and elevated C-reactive protein, were significant. Echocardiogram revealed a normal cardiac function. Imaging studies suggested the existence of bilateral pleural effusions and multiple costal fractures (Figure 1).

**Figure 2:**
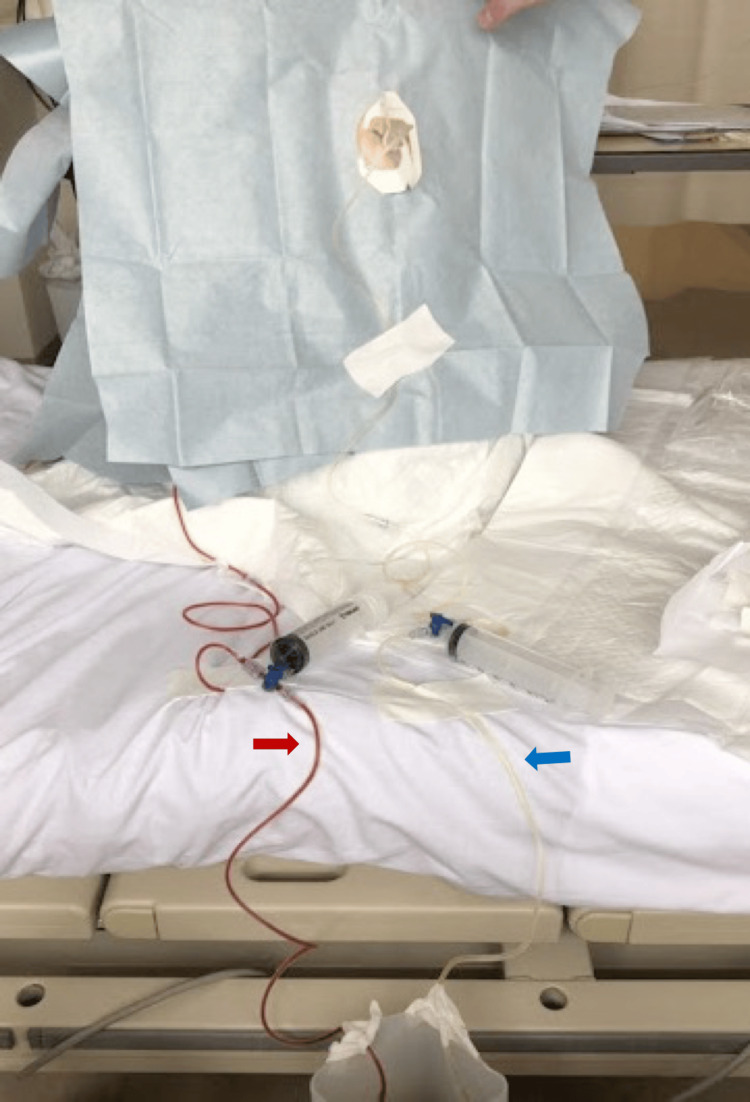
Bilateral pleural thoracentesis from the posterior view of the patient The red arrow indicates the hemorrhagic pleural effusion in the left lung, and the blue arrow points to the exudate pleural effusion in the right lung.

There were a few lesions consistent with pneumonia, but most of the lesions were attributed to pleural effusions. We performed bilateral thoracentesis. According to Light’s criteria, the fluid from the right-sided thoracentesis was identified as an exudate effusion [4]. Although the bacterial culture, the Gram stain and pus in the fluid were negative, the pH in the fluid was 7.1, and glucose in the fluid was 40 mg/dl. We inferred that the exudative pleural effusion was a complicated parapneumonic effusion resulting from aspiration pneumonia. On the other hand, the left thoracentesis revealed a hemorrhagic pleural effusion (Figure 2).

Based on these findings, although we were unable to establish the patient's precise history, we concluded that the patient developed a left-sided hemorrhagic pleural effusion resulting from costal fractures after a fall, and a right-sided complicated parapneumonic effusion resulting from aspiration pneumonia. We placed 24 Fr double-lumen drainage tubes between the mid to anterior axillary line in the fifth intercostal space above the ribs bilaterally while continuing the treatment with ampicillin-sulbactam 3 g every six hours for two weeks and rehabilitation for dysphagia. On the sixth hospital day, we removed the tubes as the patient's condition had significantly improved. He was discharged back to the nursing facility on the 17th hospital day.

## Discussion

Studies indicate that approximately 95% of etiologies in patients with bilateral pleural effusions can be attributed to a single disease [1,2]. However, in about 5% of cases, bilateral pleural effusions result from different etiologies concurrently, a condition known as Contarini's syndrome [1-3]. There are no standard criteria for considering bilateral thoracentesis in patients with bilateral pleural effusions. Some studies suggest considering bilateral thoracentesis in patients presenting with specific risk factors: elderly age, immunocompromised status, a disparity in the size of pleural effusions, lobulated effusions, differing attenuation values on CT scans for each effusion, atypical clinical findings suggestive of heart failure (such as fever and chest pain), unilateral resolution of pleural effusion after treatment, bilateral pneumonia, malignancy, and systemic disease [1,2]. This patient exhibited several risk factors: advanced age and a significant size difference between the pleural effusions. Additionally, the patient's cognitive decline might also contribute to the diverse etiologies of his condition. It is supposed to establish the patient's precise history and perform a thorough physical examination in order to assess the pre-test probability of Contarini's syndrome, since the definitive decision to perform bilateral thoracentesis depends on the physician's level of suspicion. However, this can occasionally be impossible in elderly patients with comorbidities. This case emphasizes the importance of considering bilateral thoracentesis with a low threshold, particularly in geriatric patients with risk factors.

## Conclusions

Although rare, bilateral pleural effusions can occur simultaneously due to different etiologies, known as Contarini's syndrome. Physicians should consider bilateral thoracentesis, especially in elderly patients with bilateral pleural effusions who have risk factors associated with Contarini's syndrome.
